# Early foraging life: spatial and temporal aspects of landmark learning in the ant *Cataglyphis noda*

**DOI:** 10.1007/s00359-018-1260-6

**Published:** 2018-04-20

**Authors:** Pauline Nikola Fleischmann, Wolfgang Rössler, Rüdiger Wehner

**Affiliations:** 1 Behavioral Physiology and Sociobiology (Zoology II), Biozentrum, University of Würzburg, Am Hubland, 97074 Würzburg, Germany; 2 Brain Research Institute, University of Zürich, Winterthurerstrasse 190, 8057 Zurich, Switzerland

**Keywords:** Learning walks, Landmark guidance, Experience-dependent behavior, Cue-conflict experiments, Desert ants

## Abstract

Within the powerful navigational toolkit implemented in desert ants, path integration and landmark guidance are the key routines. Here, we use cue-conflict experiments to investigate the interplay between these two routines in ants, *Cataglyphis noda*, which start their foraging careers (novices) with learning walks and are then tested at different stages of experience. During their learning walks, the novices take nest-centered views from various directions around the nest. In the present experiments, these learning walks are spatially restricted by arranging differently sized water moats around the nest entrance and thus, limiting the space available around the nest and the nest-feeder route. First, we show that the ants are able to return to the nest by landmark guidance only when the novices have had enough space around the nest entrance for properly performing their learning walks. Second, in 180° cue-conflict situations between path integration and landmark guidance, path integration dominates in the beginning of foraging life (after completion of the learning walks), but with increasing numbers of visits to a familiar feeder landmark guidance comes increasingly into play.

## Introduction

Returning to the nest after foraging is essential for all central place foragers. Ants, prime examples of central place foragers, cope with this task by pursuing several navigational strategies to return to their nest after searching for food (Wehner 2008; Graham 2010; Zeil 2012; Knaden and Graham 2016; Graham and Philippides 2017). *Cataglyphis* desert ants primarily rely on path integration (PI) involving a celestial compass for determining directions (Wehner and Müller 2006) and a stride integrator (Wittlinger et al. 2006) as well as an optic flow meter (Ronacher and Wehner 1995; Pfeffer and Wittlinger 2016) for gauging distances travelled. In addition, they make heavy use of landmark information. At the beginning of their forager career, they learn landmark configurations around the nest entrance by performing well-structured learning walks when leaving the nest for the first time (Fleischmann et al. 2016). These learning walks include characteristic turns (Fleischmann et al. 2017). The most conspicuous ones in *Cataglyphis noda* are pirouettes (also described for *Cataglyphis bicolor*: Wehner et al. 2004, and *Ocymyrmex robustior*: Müller and Wehner 2010) during which the ants look back to the nest, presumably to take goal-centered snapshots from various locations around the nest (Graham et al. 2010; Müller and Wehner 2010; Fleischmann et al. 2017; Grob et al. 2017; Fleischmann et al. 2018). The learning walks increase in size with experience, i.e., in subsequent trips, the ants move further away from the nest entrance and cover larger areas (Wehner et al. 2004; Stieb et al. 2012; Fleischmann et al. 2016, 2017) as do flying hymenopterans during learning flights (honeybees: Capaldi et al. 2000; Degen et al. 2015; bumblebees: Osborne et al. 2013). Here, we investigate how ants acquire spatial information at the beginning of their foraging career. We hypothesize that they start by relying on PI, and that with increasing outdoor experience, they gradually acquire landmark knowledge of their nest surroundings. In this line, we further hypothesize that when displaced to locations at which the ants have never been before, and at which the steering commands by PI and landmark guidance (LG) are set into conflict, the ants would gradually switch from relying on the former to using the latter. In particular, we investigate the spatial and temporal characteristics of this process, i.e., how the acquisition of landmark information depends on the space available to the ants for performing their learning walks and on the number of foraging journeys.

## Materials and methods

### Test animals, study site, and general experimental procedure

Experiments were performed with *C. noda* (Brullé 1832) in the summers of 2015 and 2016 in the Schinias National Park near Marathon, Greece, using three nests located in different clearings in the surrounding pine forest. The trees offered prominent skylines with natural landmarks. Experienced foragers were marked with one color for 3 days before experiments started. After this period, all unmarked ants were considered to be naïve (“novices”). They were caught and marked with a unique multi-color code using car paint (Motip Lackstift Acryl, MOTIP DUPLI GmbH, Haßmersheim, Germany). All visits of these individually identifiable ants at the feeder (distance between nest entrance and feeder was always 5.0 m) were registered. After a specific number of visits (depending on the experimental paradigm), ants were caught for testing and released at different release points in the clearing (depending on the experimental paradigm). To facilitate the recording of the ants’ search paths, a grid using cord was constructed (about 20 m × 20 m, mesh width: 1 m). We recorded the ant’s path with pen and paper true to scale until it returned into the nest or for a maximum of 5 min.

### Free-field experiment

In the free-field experiment (carried out at nest 1 in 2015), ants could freely explore the nest’s surroundings and forage without any spatial restriction. An artificial feeder was set up at 5.0 m east of the nest entrance and every visit of an ant was noted. After ten visits, the ant was captured at the feeder and released there. Its homebound run was recorded. The same ant was allowed to return to the feeder and bring home a food item from the feeder once before being captured again and released at one of the other release points (5.0 m south, west, or north from the nest entrance). This procedure was repeated until the ant had been tested at every release point. Ten ants were tested at all four release points. In addition, 16 novices were tested when they occurred for the first time outside the nest and had not yet performed their learning walks, each at one release point. Since it was not possible to train the ants only in the direction of the feeder and catch them at their first feeder visit, a glass channel (height: 0.3 m, width: 0.3 m, length: 5.3 m, feeder 5.0 m north from the nest entrance) was installed at another nest (nest 2). Ants could explore the area within the channel and were captured when they visited the feeder for the first time. Ten ants were tested, each at one of three release points (5.0 m east, south, or west from the nest entrance).

### Moat experiment

**Fig. 1:**
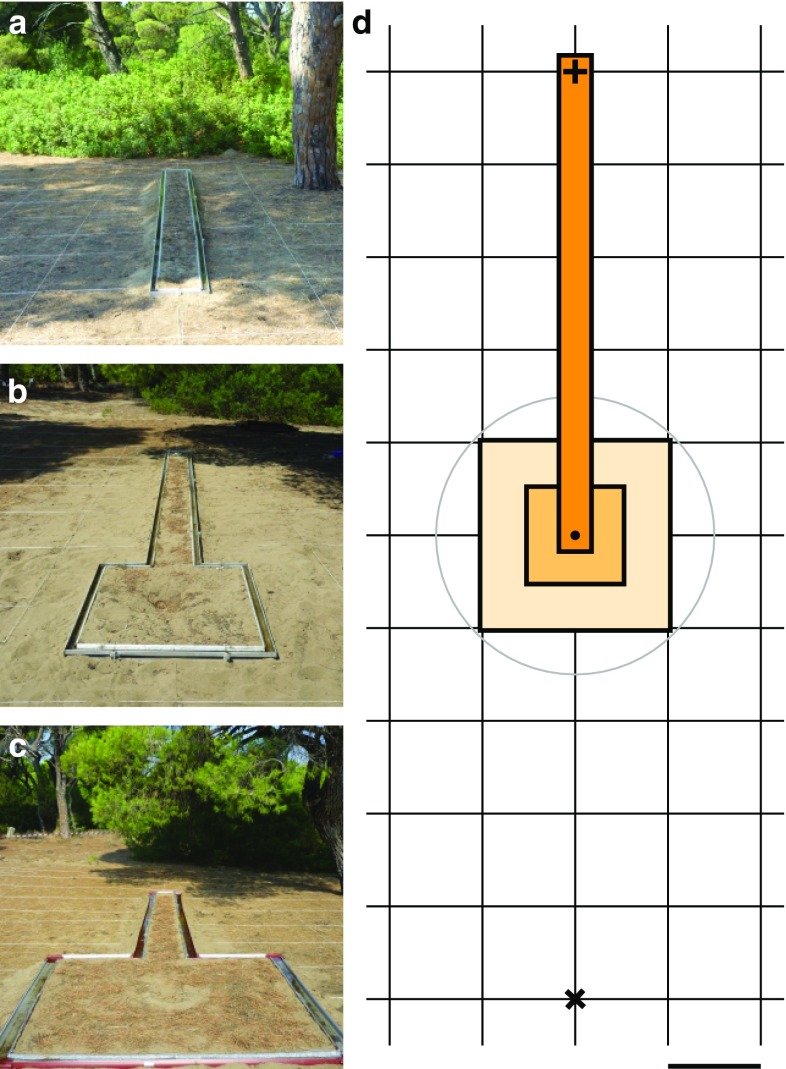
Moat setup. Three differently sized water moats (**a** moat setup 1, **b** moat setup 2, and **c** moat setup 3) offered ants space to perform learning walks around their nest entrance before being trained to a feeder and released in the test field later on. **d** Scheme illustrates the spatial relations: the distance between nest entrance (shown as black dot) and feeder (shown as black +) was always 5 m. The release point (shown as black ×) was 5 m of the nest entrance in the opposite direction of the feeder. Mesh size of the test grid was 1 m (shown by the scale bar). The runway had a width of 0.3 m and a length of 5.3 m. Ants trained in the moat setup 1 could only walk in the dark orange area. When moat setup 2 was installed, ants had, additionally, 1 m^2^ around the nest entrance (light orange), and with moat setup 3, they had 4 m^2^ (completely colored area). The grey circle indicates the “nest area”. In tests, ants in all setups could reach this area without any restrictions; and if they did, they were counted as returning successfully to the nest

Since we were not sure whether the walks in the glass channel might influence the ants’ navigational performances by altering the panorama or reflecting light, we restricted the area around the nest entrance using a moat filled with seawater. Three differently sized setups were used to test the influence of space available to perform learning walks on homing success during testing in 2016. At nest 2, we first installed a moat that offered the ants only a narrow runway (moat setup 1: width: 0.3 m, length: 5.3 m, feeder 5.0 m north of the nest entrance, Fig. 1a). To offer ants more space for performing learning walks, two other setups were used. At nests 2 and 3, a water moat was installed that offered 1 m^2^ free area around the nest entrance (moat setup 2, Fig. 1b). At nest 3, a larger setup offering 4 m^2^ free area around the nest entrance was set up using gutters (moat setup 3, Fig. 1c). The feeder was placed 5.0 m west of the nest entrance at nest 3. These three setups offered different amounts of space available to the ants to perform their learning walks (Fig. 1d). In moat setup 1, the ants could move in all directions 15–20 cm away from the nest entrance. In this way, novices could only perform their very first learning walks (Fleischmann et al. 2016, 2017) without stumbling upon water moat restrictions. In moat setups 2 and 3, the ants could walk 50–70 cm (cf. learning walk category 2 in Fleischmann et al. 2016), and 100–140 cm in each direction, respectively, before they reached the water moat. If an ant hit the moat and stumbled into the water, it immediately returned to the nest.

To test the influence of experience gained over time, we applied three different testing regimes. Ants were assigned to one of the three experimental test groups. The first experimental group of ants was tested multiple times sequentially after different numbers of visits at the feeder [six full-vector tests every second feeder visit until their fifth test, and, additionally, for the sixth time after the 16th feeder visit (FV I–VI), and subsequently one zero-vector test (ZV)]. Ants of the second experimental group were tested once for the first time after their tenth feeder visit (FV 10+) and once as zero-vector ant (ZV 10+). Ants of the third group were only tested once after their first feeder visit (FV 1st). All experimental regimes were performed in all three setups (for details: see Table 1).

**Table 1:** Experimental regimes

Setup	Regime	*n*	Feeder visit no.																	
			1	2	3	4	5	6	7	8	9	10	11	12	13	14	15	16	17	18
Moat setup 1	Multiple tests	10	**FV I**	N	FV II	N	FV III	N	FV IV	N	FV V	N	N	N	N	N	N	**FV VI**	N	**ZV**
	After 10th visit	21	N	N	N	N	N	N	N	N	N	**FV 10**+	N	**ZV 10**+	–	–	–	–	–	–
	After first visit	33	**FV 1st**	–	–	–	–	–	–	–	–	–	–	–	–	–	–	–	–	–
Moat setup 2	Multiple tests	7	**FV I**	N	FV II	N	FV III	N	FV IV	N	FV V	N	N	N	N	N	N	**FV VI**	N	**ZV**
	After 10th visit	16	N	N	N	N	N	N	N	N	N	**FV 10**+	N	**ZV 10**+	–	–	–	–	–	–
	After first visit	32	**FV 1st**	–	–	–	–	–	–	–	–	–	–	–	–	–	–	–	–	–
Moat setup 3	Multiple tests	21	**FV I**	N	FV II	N	FV III	N	FV IV	N	FV V	N	N	N	N	N	N	**FV VI**	N	**ZV**
	After 10th visit	18	N	N	N	N	N	N	N	N	N	**FV 10**+	N	**ZV 10**+	–	–	–	–	–	–
	After first visit	33	**FV 1st**	–	–	–	–	–	–	–	–	–	–	–	–	–	–	–	–	–

Ants were either tested multiple times (six full-vector tests FV I–VI and one zero-vector test ZV) or once as full-vector (FV 1st and FV 10+) ants

Ants tested only once were either tested after their first (FV 1st) or their tenth (FV 10+) feeder visit. FV 10+ ants were, additionally, tested as zero-vector (ZV 10+) ants as were the multiple tested ants. Between tests, ants were allowed to return to their nest (N) from the feeder taking a food item home

The results of the tests marked bold are presented in the figures and compared statistically

The general experimental procedure was the same in all three moat setups. After all foragers had been color-marked during three successive days, an ant leaving the nest for the first time (a “novice”) was captured at the nest entrance and marked individually. When it reached the feeder in the following days and took a food item, this event was counted as the first feeder visit. Ants were tested at different stages of experience over time depending on their experimental group (for details: see Table 1). Test ants were transferred to the release point in a dark tube and released within a plastic ring (diameter: 16 cm). They were offered a food item and were released when they had taken the food item or after 5 min. Their paths were recorded for 5 min or until they reached the moat.

### Data analysis and statistics

The homing success rate was measured in each experimental group by counting what proportion of the homing ants entered the so-called “nest area”, an area of 1.5 m around the nest entrance, within 5 min. If the ants reached this area, they usually found back to the nest entrance or touched the water channel a few moments later. We compared the results of the different experimental groups using Fisher’s exact test (two-sided) with Bonferroni correction. The significance level was *α* = 0.05. This test was also used for the comparison of the proportion of ants that took or refused food items when they were released.

The protocol sheets of the ants’ search paths were scanned and then processed using GIMP 2.8.10. Examples of ants’ paths were copied using the pencil tool (size 5.0).

The distance between release point and turning point (the point where an ant stopped to follow one direction) was measured. The turning point was determined by selecting a circle using the ellipse select tool and expanding it from the center at the release point until the ant’s path followed the circle or touched it and reversed towards the inside of the circle. The distance between release point and turning point was the radius of the circle. We compared the median distances of the first and sixth tests of the sequentially tested ants pairwise within the different experimental setups (i.e., moat setup 1, 2, and 3) using the Mann–Whitney *U* test with a significance level of *α* = 0.05. The same statistical test was performed for the first tests of ants tested once after their first feeder visit and the first tests of ants tested after their tenth visit at the feeder. All statistical tests were performed with Matlab R2014b (MathWorks, Inc., Natick, MA, USA).

Figures were edited with Corel Draw X6 (Corel Corporation, Ottawa, ON, Canada).

## Results

### Free-field experiment: only free-field experienced foragers return to the nest area

**Fig. 2:**
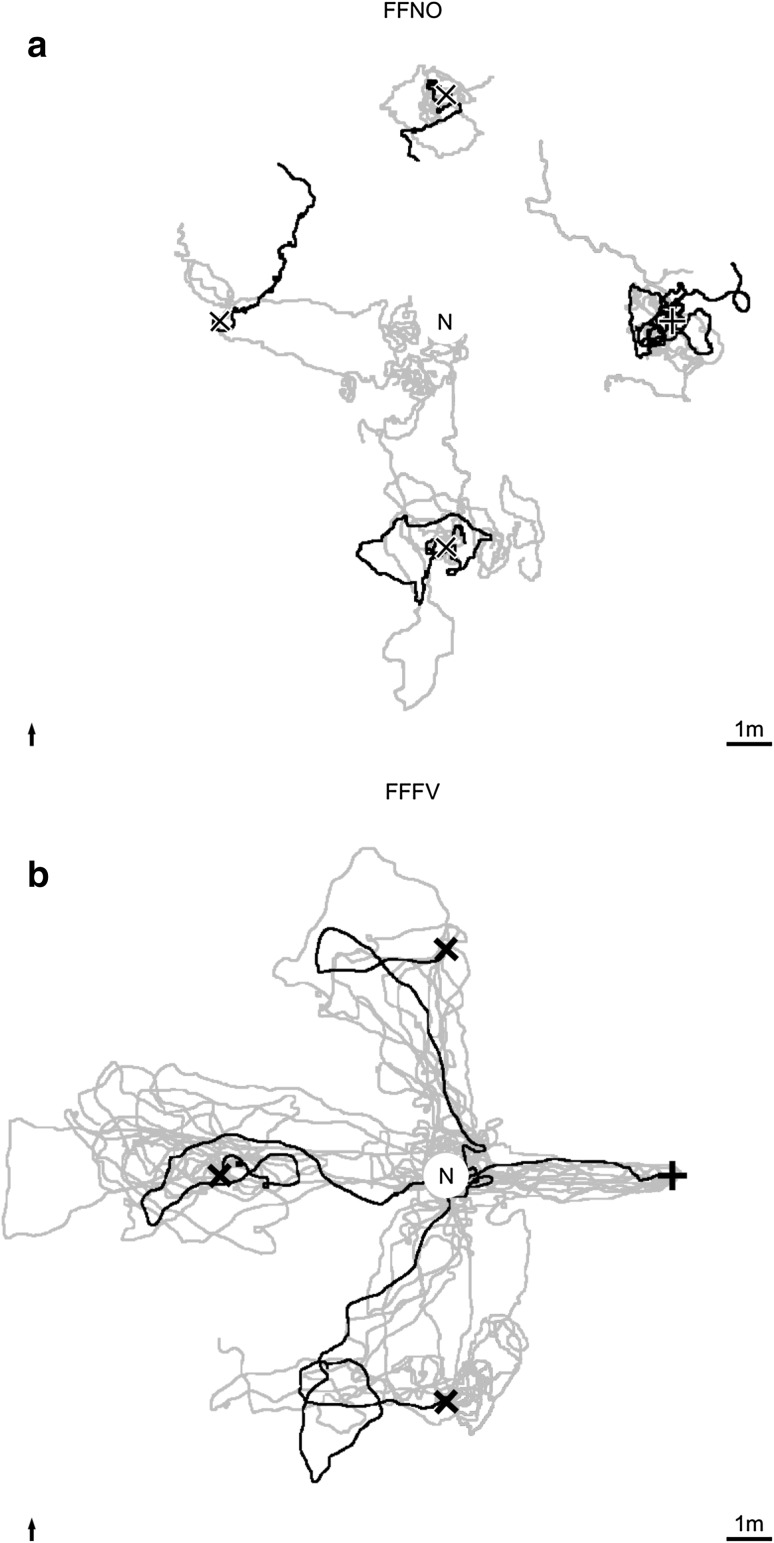
Recorded paths of novices (FFNO) (**a**) and experienced free-field foragers (FFFV) (**b**). The nest entrance (N) was located in the middle of the test field (20 m × 20 m, scale bar: 1 m). The feeder (shown as a black cross) was 5 m east of the nest entrance (the black arrow points towards north). **a** Novices (*n* = 16) were released at four different release points (i.e., feeder in the east and 5 m north, south, and west of the nest entrance shown as black ×s). Each novice was tested only once, i.e., each bold trajectory refers to a separate individual chosen randomly to show exemplarily the paths at each release point. **b** Experienced foragers (*n* = 10) were caught at the feeder and released at all four release points after visiting the feeder. The black paths show all four tests of one individual randomly chosen

**Fig. 3:**
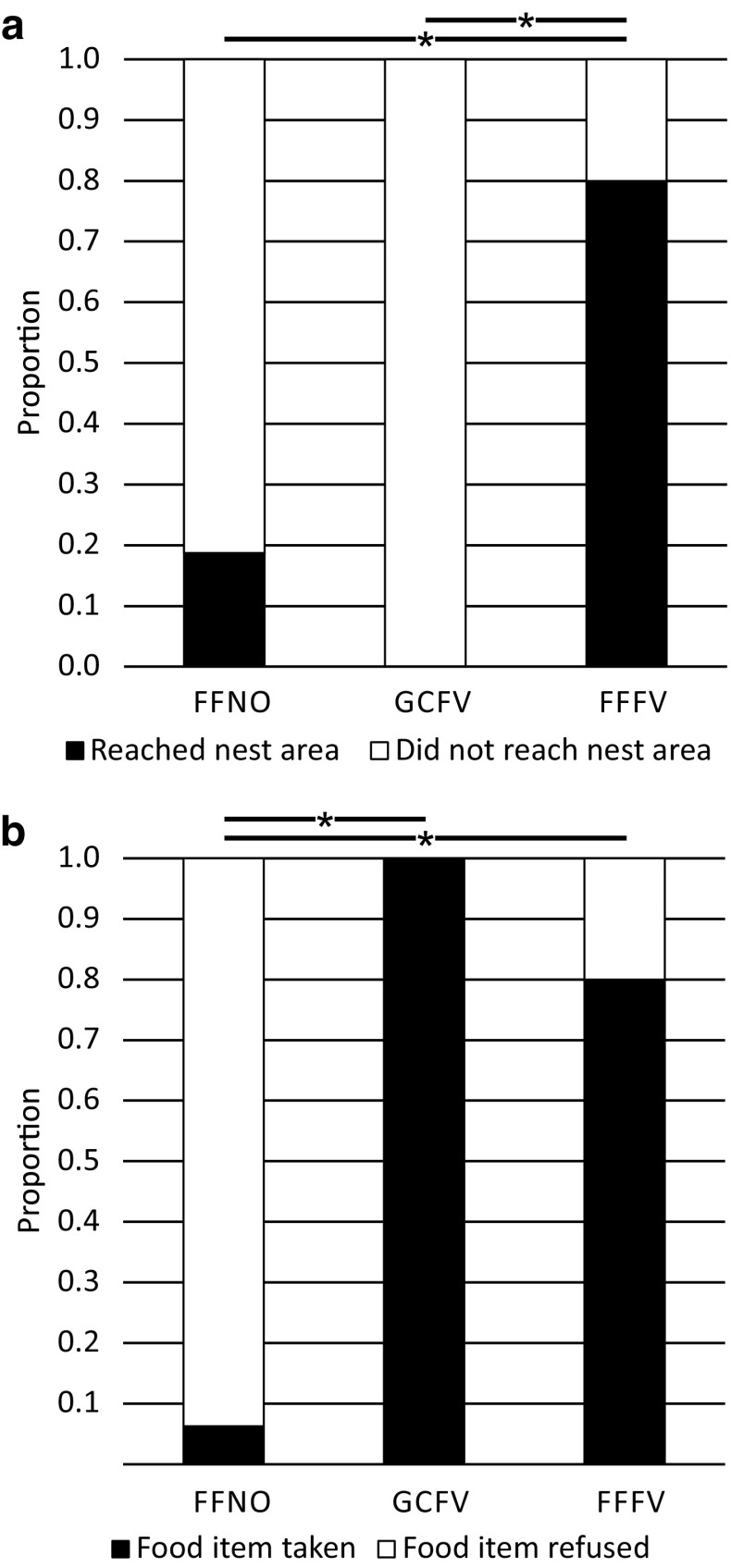
Foraging success during first or only test from a novel release point. **a** Proportion of ants reaching the nest area within 5 min. **b** Proportion of ants that took a food item when being released in the test field. Asterisks indicate significant differences between the groups when compared pairwise using Fisher’s Exact test with Bonferroni correction (*α* = 0.05, after correction 0.0167, free-field novices (FFNO): *n* = 16, glass-channel full-vector ants (GCFV): *n* = 10, free-field full-vector ants (FFFV): *n* = 10)

Free-field novices (FFNOs) captured at the nest when leaving it for the first time and transferred to release points at 5 m distance, usually did not find their way back to the nest (Fig. 2a). Only 3 out of 16 ants reached the nest area (defined as a circle of 1.5 m radius around the nest entrance) within 5 min (Fig. 3a), and only one of them actually entered the nest entrance within 5 min. A quarter (4 out of 16) of FFNOs did not move to search for the nest, but hid under grass and pine needles. In contrast, free-field full-vector (FFFV) foragers captured at the feeder 5 m east of their nest entrance (after more than ten feeder visits) were usually able to return to the nest (Fig. 2b). When released at the feeder all ants immediately returned to the nest carrying a food item (median duration: 61 s, ranging from 35 to 137 s, *n* = 10, Figs. 2b, 3). FFFV ants were also successful in homing when released in the other cardinal directions of the nest at 5 m distance (Fig. 2b). In 26 out of 30 tests (*n* = 10, each ant tested from north, south, and west), the ants reached the nest area, and in 20 out of 30 tests, they actually entered the nest entrance within 5 min. Usually, released ants followed their PI home vectors first and switched to landmark-guided navigation after a few meters (measured as the distance between release and turning point, i.e., the point where an ant stopped to follow vector direction: northern release point: 3.3 ± 1.9 m, southern release point: 3.6 ± 2.4 m, and western release point: 2.2 ± 1.3 m, median ± IQR). Overall, the release of naïve ants and experienced foragers at four different release points showed that the homing success differed drastically. Significantly more FFFVs returned to the nest than FFNOs did (Fig. 3a; for statistical details, see below). Since it was not possible to train new ants to the feeder in the free field in a way that they only got to know the way to the feeder and no other direction around the nest, a glass channel was installed to guide the ants in one direction but allowing them to view the whole panorama. Glass-channel full-vector ants (GCFVs, *n* = 10) captured at the feeder 5 m north of the nest entrance after their first feeder visit, i.e., after they had picked up a food item at the feeder, and released at one of the three release points (east, south, or west of the nest entrance) did not return to the nest. None of them entered the nest area (Fig. 3a), but they followed their PI vectors in a straight line for 4.2 ± 2.4 m (median ± IQR, ranging from 2.1 to 6.0 m) and subsequently started a systematic search for their nest. Therefore, only FFFVs were able to return home from all directions, resulting in a significant difference in homing success between those foragers and both FFNO ants as well as first visitors at the feeder in the glass channel (GCFV) (Fig. 3a, Fisher’s exact test with Bonferroni correction: FFFV (*n* = 10) versus FFNO (*n* = 16): *p* < 0.0167; FFFV versus GCFV (*n* = 10): *p* < 0.0167; GCFV versus FFNO: *p* = 0.2616). However, to double check whether GCFVs in the glass channel were actual foragers and not naïve ants lost in the channel, we compared their willingness to pick up a food item after being released with those of FFFVs and FFNOs. Both GCFVs and FFFVs took significantly more often food items than novices did before being tested [Fig. 3b, Fisher’s exact test with Bonferroni correction: FFFV (*n* = 10) versus FFNO (*n* = 16): *p* < 0.0167; FFFV versus GCFV (*n* = 10): *p* = 0.4737; GCFV versus FFNO: *p* < 0.0167].

In summary, these results first show that foragers rely on their path integrator from their first feeder visit onward. Second, experienced foragers can return from all directions to the nest even if their home vector initially leads them in another direction. However, they cannot return from release points at which they have never been before when they have been restricted by a glass channel while performing their learning walks. This raises two main questions to be answered next: (1) how much space do the ants need for performing their learning walks around the nest entrance, to be able to successfully return home by landmark guidance (LG), and (2) does their landmark-based homing success improve with increasing numbers of feeder visits?

### Moat paradigm: both spatial experience and experience over time are needed for successful homing

To answer the above questions and to test under what spatial conditions (size of area available for learning walks) and what temporal conditions (number of feeder visits) LG is able to override the dictates of the path integrator (PI), we applied the following test paradigms. Ants were trained in three differently sized moat setups (moat setups 1, 2, and 3, Fig. 1). They were captured at the feeder (F)—hence full-vector (FV) ants—were released at a location (release point R) that was at the same distance (5 m) from the nest (N), but in the opposite direction, so that the F → N direction was 180° apart from the R → N direction. Hence, at R the PI home vector pointed in the direction opposite to the nest direction (maximal cue conflict). Ants were either tested multiple times (FV I–FV VI), once after their first feeder visit (FV 1st), or after their tenth feeder visit (FV 10+). Ants that were captured and tested as FV ants after several feeder visits were, additionally, captured at the nest entrance and tested as zero-vector ants (ZV and ZV 10+, respectively).

**Fig. 4:**
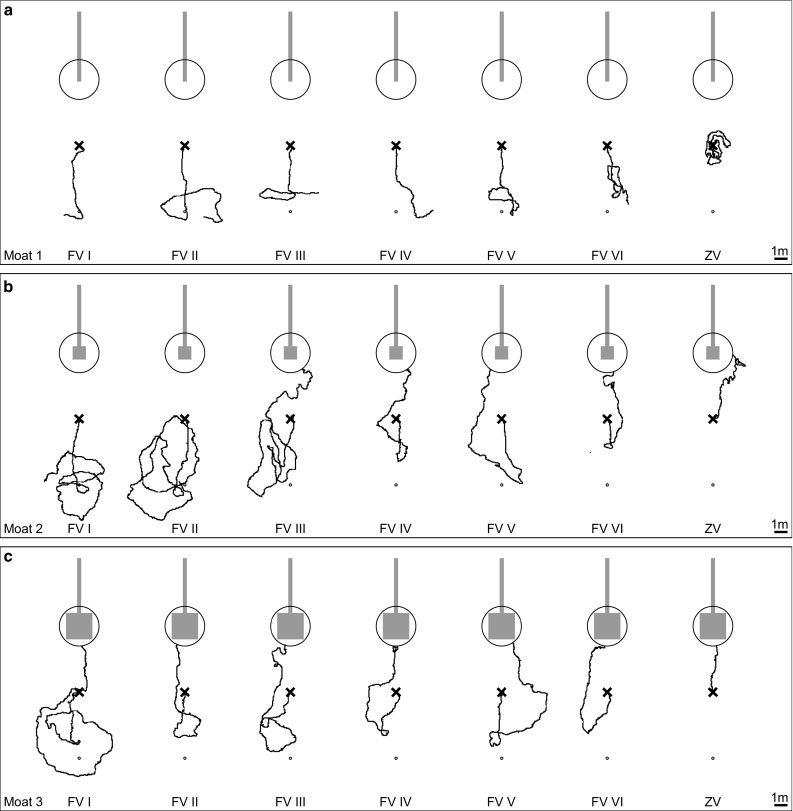
Examples of recorded paths of ants tested multiple times and trained in three different setups (**a** moat setup 1, **b** moat setup 2, and **c** moat setup 3). Setups are shown true to scale in grey. The large circle (radius: 1.5 m) includes the nest area in which the nest entrance is located in the middle. The release point is shown as a black × and the fictive nest position of the home vector as a small circle. The PI home vector points in the anti-nest direction. Paths of ants were recorded for 5 min or until ants reached the nest area. Each ant was tested six times as full-vector ant (FV I–VI) and afterwards once as zero-vector ant (ZV)

**Fig. 5:**
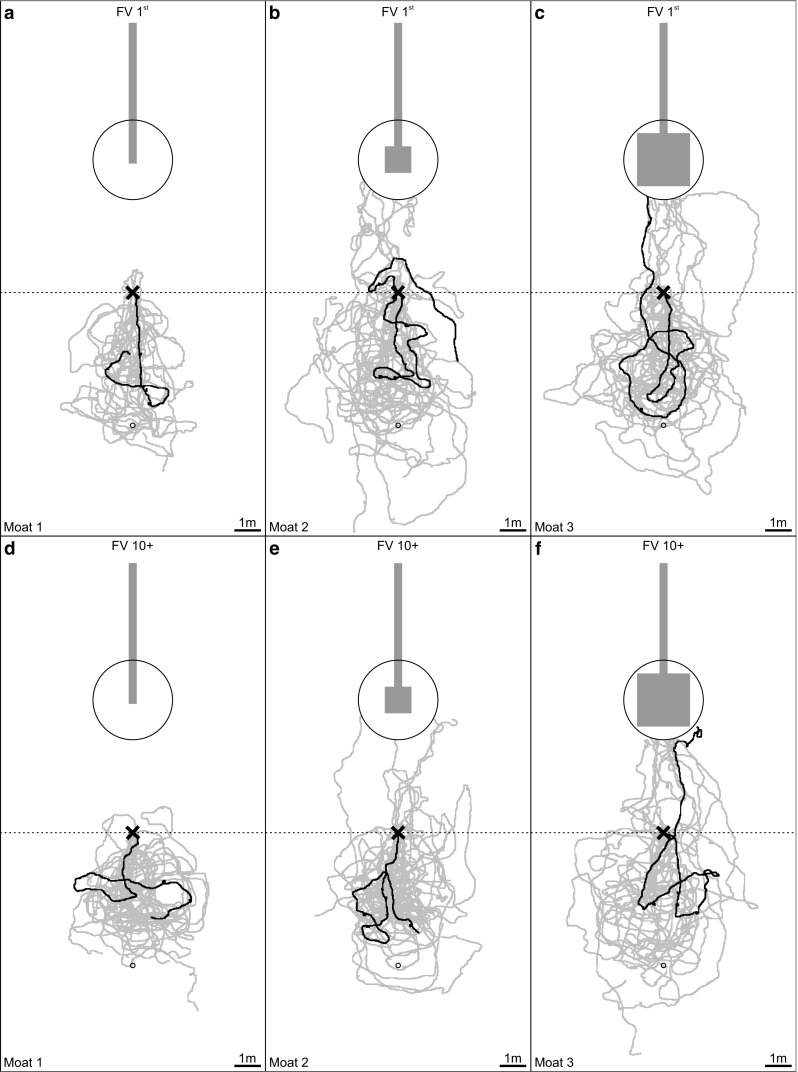
Recorded paths of FV ants tested once after their first (FV 1st: **a**–**c**) or tenth (FV 10+: **d**–**f**) feeder visit. Setups are shown true to scale in grey (**a** and **d**: moat setup 1, **b** and **e**: moat setup 2, **c** and **f**: moat setup 3). Each subfigure includes 15 examples, which were randomly chosen from all ants tested. One example is highlighted in black. For further conventions, see Fig. 4

**Fig. 6:**
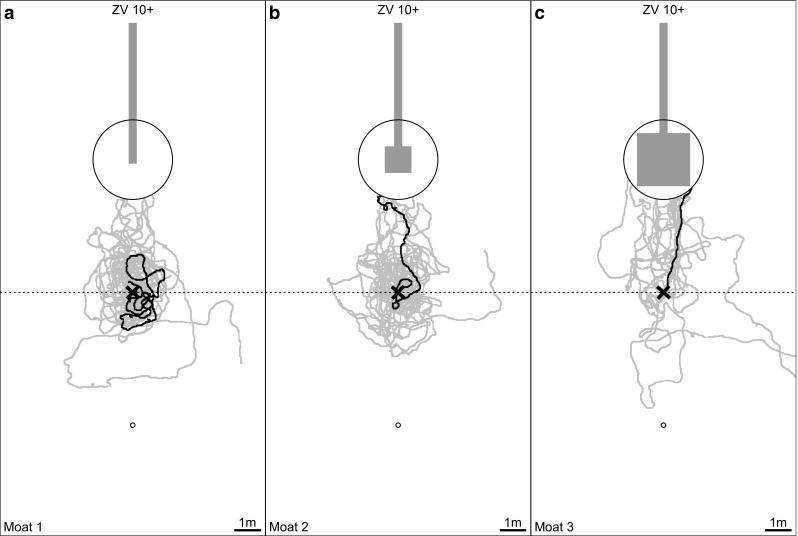
Recorded paths of ZV 10+ ants trained in three different setups (**a** moat setup 1, **b** moat setup 2, and **c** moat setup 3). For further conventions, see Fig. 5. The examples highlighted in black show the paths of the same ants as in Fig. 5d–f

In these test paradigms, we assumed that the homing success rate would increase with the area that was available for performing learning walks. We also assumed that ants displaced for the first time (FV I and FV 1st) would fully rely on their path integrator and run in the direction away from home, but with increasing numbers of visits to the feeder would stop following the home vector earlier and start to search for the nest (FV II–FV VI and FV 10+ ants). Furthermore, we expected more ants to return to the nest area when being tested as ZV ants (ZV and ZV 10+) than as FV ants. The results of our experiments confirmed all three assumptions, and the main results are clear when simply looking at the recorded search paths of the tested ants. In a nutshell, ants only returned to the nest area if they had had space to perform learning walks (moat setups 2 and 3, versus moat setup 1, Figs. 4, 5). Ants tested multiple times followed their home vector fully when tested for the first time (FV I in Fig. 4 a moat setup 1, b moat setup 2, and c moat setup 3), but stopped following the home vector earlier with increasing experience, i.e., with an increasing number of feeder visits and tests (FV II–FV VI in Fig. 4). The same effect could be observed when comparing ants tested once after their first feeder visit (FV 1st) with ants tested once after their tenth feeder visit (FV 10+) (Fig. 5, moat setup 1: a versus d, moat setup 2: b versus e, and moat setup 3: c versus f). More ants reached the nest area when tested as ZV ants (Fig. 4 ZV and Fig. 6) than when tested as FV ants.

In the next sections, we take a closer look at the effect of the spatial and temporal experiences that the ants could gain while performing their learning walks and their first foraging journeys.

#### Spatial aspects

**Fig. 7:**
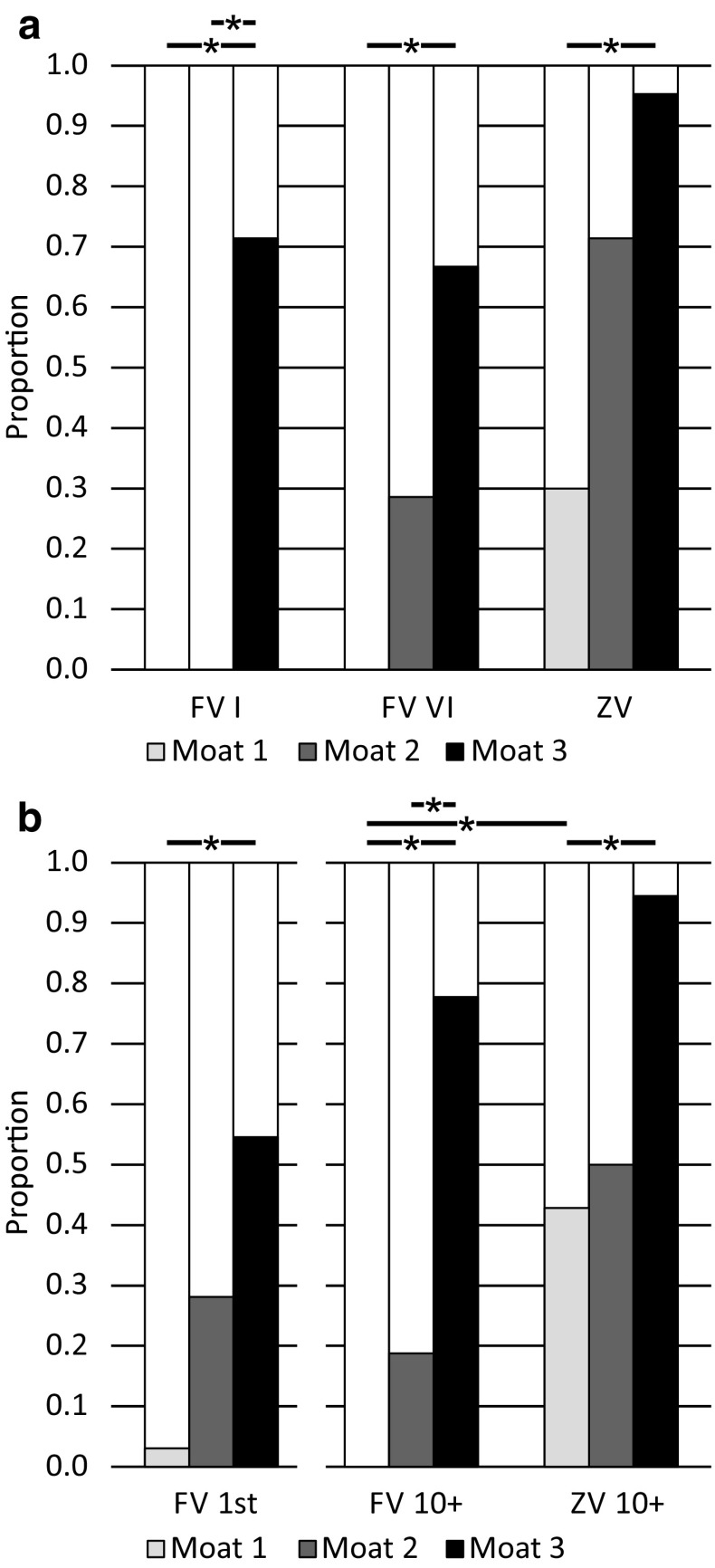
Homing success of ants trained in three differently sized moat setups. **a** Proportion of ants reaching the nest area tested multiple times (moat setup 1: *n* = 10, moat setup 2: *n* = 7, moat setup 3: *n* = 21). **b** Proportion of ants reaching the nest area tested once either after their first (FV 1st : moat setup 1: *n* = 33, moat setup 2: *n* = 32, moat setup 3: *n* = 33) or tenth (FV 10+: moat setup 1: *n* = 21, moat setup 2: *n* = 16, moat setup 3: *n* = 18) feeder visit. FV 10+ ants were, additionally, tested as zero-vector ants (ZV 10+) after two additional feeder visits. Asterisks indicate significant differences between the groups when compared pairwise using Fisher’s exact test with Bonferroni correction (*α* = 0.05, after correction in **a** 0.0028 and in **b** 0.0042). Experimental groups were compared across setups at each test. Furthermore, the proportions of the same ants tested more than once were compared

Ants trained with the moat setup 1 always followed their home vector and subsequently started a systematic search (Figs. 4a, 5a, d, 7). Only one of a total of 64 ants trained this way (FV 1st : *n* = 33, FV 10+: *n* = 21, and FV ants tested multiple times, i.e., FV I and FV VI: *n* = 10) reached the nest area during testing. In contrast, some ants trained in moat setup 2 (Figs. 4b, 5b, e, 7) and always more than half of the ants trained in moat setup 3 (Figs. 4c, 5 c, f, 7) reached the nest area within 5 min. Overall, the proportion of ants homing successfully was higher, the larger the training setup had been (Fig. 7). This increase in homing success was significant when ants had been trained in the large moat setup 3 compared to ants trained in the moat setup 1 [Fisher’s exact test with Bonferroni correction: ants tested multiple times, moat setup 1 (*n* = 10) versus moat setup 3 (*n* = 21), for each test (FV I, FV VI, ZV): *p* < 0.0028; ants tested once either after their first (FV 1st) or tenth (FV 10+) visit, the latter, additionally, as zero-vector ants (ZV 10+): for each test (moat setup 1 FV 1st (*n* = 33) versus moat setup 3 FV 1st (*n* = 33), moat setup 1 FV 10+ (*n* = 21) versus moat setup 3 FV 10+ (*n* = 18), and moat setup 1 ZV 10+ (*n* = 21) versus moat setup 3 ZV 10+ (*n* = 18)] *p* < 0.0042). Furthermore, significantly more ants reached the nest area when comparing FV I of moat setup 2 with moat setup 3, and FV 10+ of moat setup 2 with moat setup 3 (Fisher’s exact test with Bonferroni correction: FV I moat setup 2 (*n* = 7) versus moat setup 3 (*n* = 21): *p* < 0.0028; FV 10+ moat setup 2 (*n* = 16) versus moat setup 3 (*n* = 18): *p* < 0.0042). All other pairwise comparisons between the experimental groups in differently sized setups revealed no significant differences, although there is a tendency of more ants to reach the nest area when the training setup offers more room to perform learning walks during training. Ants that had visited the feeder multiple times before the tests were, additionally, tested as ZV ants (Fig. 4: ZV, and Fig. 6: ZV 10+). The homing success rate of ants without any PI vector information was always higher than that of ants tested as FV ants (Fig. 7). This is due to the fact that, after release, the FV ants first follow their PI instructions (see section “Temporal aspects”). However, the better homing performance of the ZV ants was only significant when comparing FV 10+ with ZV 10+ of moat setup 1 trained ants (Fisher’s exact test with Bonferroni correction: moat setup 1 (*n* = 21) FV 10+ versus ZV 10+: *p* < 0.0042). The difference between the behavior of FV and ZV ants is not only borne out statistically by comparing the homing success rates, but also shown in the searching behavior. Moat setup 1 trained ants immediately started systematic searching around the release point (Fig. 4a ZV, Fig. 6a), whereas moat setup 3 ants usually approached the nest directly in a straight line (Fig. 4c ZV, Fig. 6c). Ants trained in moat setup 2 showed an intermediate behavior. Sometimes they searched systematically and sometimes they approached the nest directly (Fig. 4b ZV, Fig. 6b).

#### Temporal aspects

**Fig. 8:**
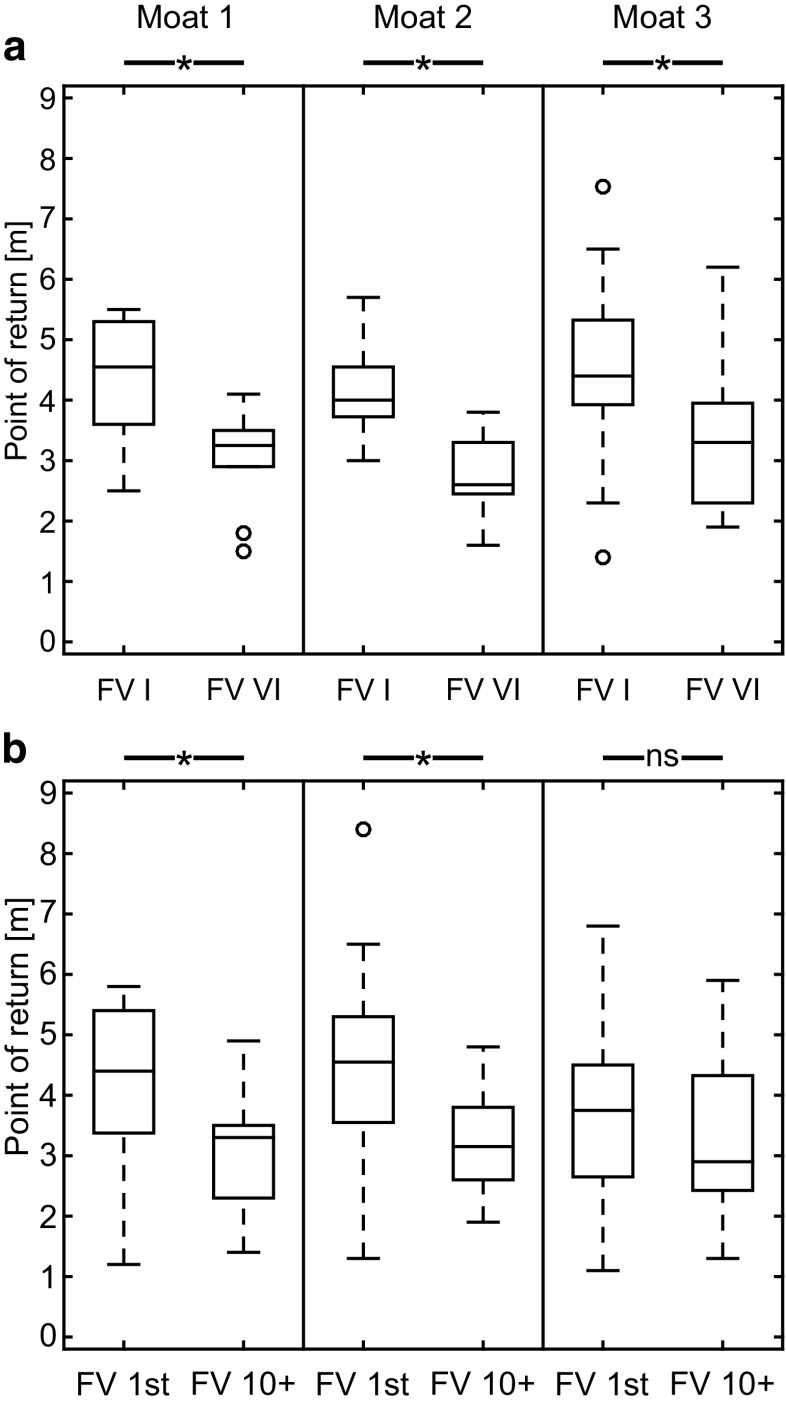
Turning points of ants during testing in different testing regimes and different experimental setups. **a** Turning points of ants that were tested after their first and subsequent visits at the feeder (FV I versus FV VI) were compared in three different moat setups [left: moat setup 1 (*n*_FV I_ = 10, *n*_FV VI_ = 10), middle: moat setup 2 (*n*_FV I_ = 7, *n*_FV VI_ = 7), right: moat setup 3 (*n*_FV I_ = 19, *n*_FV VI_ = 21)]. **b** Turning points of ants that were tested once either after their first (FV 1st : moat setup 1: *n* = 33, moat setup 2: *n* = 32, moat setup 3: *n* = 32) or tenth (FV 10+: moat setup 1: *n* = 21, moat setup 2: *n* = 16, moat setup 3: *n* = 17) feeder visit in three different moat setups (left: moat setup 1, middle: moat setup 2, right: moat setup 3). Groups were compared pairwise using Mann–Whitney *U* test (*α* = 0.05). Asterisks indicate significant differences between groups

Ants tested for the first time after their first feeder visit followed their home vectors almost completely (Fig. 8; moat setup 1: FV I (*n* = 10): 4.6 ± 1.4 m; FV 1st (*n* = 33): 4.4 ± 2.0 m; moat setup 2: FV I (*n* = 7): 4.0 ± 0.8 m; FV 1st (*n* = 32): 4.6 ± 1.7 m; moat setup 3: FV I (*n* = 19): 4.4 ± 1.2 m; FV 1st (*n* = 32): 3.8 ± 1.7 m, median ± IQR). In contrast, ants that had gained more experience before being tested stopped to follow their vectors earlier (Fig. 8; moat setup 1: FV VI (*n* = 10): 3.3 ± 0.6 m; FV 10+ (*n* = 21): 3.3 ± 1.2 m; moat setup 2: FV VI (*n* = 7): 2.6 ± 0.6 m; FV 10+ (*n* = 16): 3.2 ± 1.1 m; moat setup 3: FV VI (*n* = 21): 3.3 ± 1.6 m; FV 10+ (*n* = 17): 2.9 ± 1.8 m, median ± IQR). This shortening as shown in Figs. 4 and 5 is statistically significant in five of six pairwise comparisons (Fig. 8, Mann–Whitney *U* test: moat setup 1: FV I versus FV VI: *z* = 2.4226, *n*_FV I_ = 10, *n*_FV VI_ = 10, *p* < 0.05; FV 1st versus FV 10+, *z* = 3.7411, *n*_1st_ = 33, *n*_10+_ = 21, *p* < 0.05 moat setup 2: FV I versus FV VI: *z* = 2.6252, *n*_FV I_ = 7, *n*_FV VI_ = 7, *p* < 0.05, FV 1st versus FV 10+: *z* = 3.1082, *n*_1st_ = 32, *n*_10+_ = 16, *p* < 0.05; moat setup 3: FV I versus FV VI: *z* = 2.7529, *n*_FV I_ = 19, *n*_FV VI_ = 21, *p* < 0.05, FV 1st versus FV 10+: *z* = 0.12085, *n*_1st_ = 32, *n*_10+_ = 17, *p* = 0.2269). Some ants were not only tested once after a specific number of feeder visits, but multiple times after every second feeder visit (FV I–VI). The most abrupt shortening of the PI-guided path segment occurred between the first test (FV I) after the first feeder visit and the second test (FV II) after the third feeder visit, i.e., after the ant had experienced the F → N route once. In subsequent tests, the ants followed their PI vectors less far, but never ignored the PI vector information completely and hence never relied exclusively on LG (examples of three individual ants are shown in Fig. 4). As a result, with increasing number of feeder visits, FV ants follow their PI home vector for increasingly shorter distances, i.e., gradually increase their readiness to home by LG. Even though the ants stopped following their home vector earlier when they had performed more feeder visits before the tests, their homing success rates did not increase significantly in any experimental setup (Fig. 7a, Fisher’s exact test with Bonferroni correction: moat setup 1 (*n* = 10): FV I versus FV VI, *p* = 1; moat setup 2 (*n* = 7): FV I versus FV VI, *p* = 0.4616; moat setup 3 (*n* = 21): FV I versus FV VI, *p* = 1).

## Discussion

In the present study, we asked how spatial restriction of the area around the nest entrance and control of the number of feeder visits impede homing success of foragers when tested in a displacement experiment. The critical experimental parameters differing between test groups were, on one hand, the space available around the nest entrance to perform learning walks before testing started, and, on the other hand, the number of visits at the feeder, and how often the ants were tested. Both dimensions of experience significantly influenced the homing abilities of the ants (see Figs. 7, 8 for spatial experience and experience gained over time, respectively).

### Spatial requirements of learning walks

At the start of its foraging life, a *Cataglyphis* ant performs a sequence of learning walks, with increasing length and duration from one learning walk to another (*C. bicolor*: Wehner et al. 2004; *C. fortis*:; Stieb et al. 2012; Fleischmann et al. 2016, 2017; *C. noda*:). During these learning walks, the ant stops at different places and distances and looks back to the nest entrance (Fleischmann et al. 2017). Most likely it is during these turn-back and-look events that the ants acquire and store goal-centered panoramic images (Graham et al. 2010; Müller and Wehner 2010; Fleischmann et al. 2017; Grob et al. 2017; Fleischmann et al. 2018). The present study shows that enough space around the nest entrance is required for successfully taking the necessary panoramic views. By confining the ants’ learning walks to differently sized areas around the nest entrance, we investigated how much space the novices need in their pine forest habitat to acquire the landmark memories necessary for successful homing. A glass channel and three different water moat configurations (Fig. 1) restricted the learning walks to various degrees. The experimental setups restricted the learning walks in a way that the ants could not explore all directions around the nest entrance similarly. Ants were stopped after 15–20 cm (glass channel and moat 1), 50–70 cm (moat 2), or 100–140 cm (moat 3), respectively. When novices hit the moat and stumbled into the water, they stopped their learning walk and immediately returned to the nest (personal observation). Therefore, the experimental setups disturbed the learning walks with respect to at least three different aspects: first, novices could not explore all directions equally. Second, they could only leave the nest until reaching a certain distance, i.e., their learning walks were shorter in length and, therefore, included fewer pirouettes (the latter aspect has to be investigated in detail in future studies). Third, the smaller the area around the nest entrance, the more often ants hit the moat. This may have repeatedly frustrated them and thus led to less confidence in their knowledge about the panorama.

Only in moat setup 3 could the ants perform extensive learning walks up to 1 m distance in all directions from the nest entrance. As shown in Figs. 3a and 7, the differences in space offered to the ants for performing their learning walks led to marked differences in homing success. Ants trained in a 0.3 m-wide runway (moat setup 1 or glass channel) never returned to the nest by landmark guidance (LG) (Figs. 4a, 5a, d). Similarly, *C. fortis* novices cannot find back to the (fictive) nest entrance position guided by landmarks when they have been captured and tested after short learning walks (category 1: < 0.3 m, category 2: < 0.7 m, Fleischmann et al. 2016). In contrast, *C. noda* ants, which had free-field experience (Fig. 2b) or had performed their learning walks in the large setup (moat setup 3, Figs. 4c, 5c, f), reliably reached the nest area when displaced to a novel location. Hence, the first conclusion drawn on the basis of the results from the current experiments is that spatial restrictions of the learning walks decrease the ants’ homing success significantly. Importantly, the ants are released in the neighborhood (at a 5 m distance) of their nest located in the middle of a clearing in their pine forest habitat, but at places at which they have never been before. This is also the case when, in full-vector (FV) ants, path integration (PI) and LG systems are in conflict by indicating opposite directions. There are two possible explanations how the spatial restrictions of learning walks may impede the learning of the landmark panorama: on the one hand, the spatially restricted learning walks enable only to take snapshots close to each other resulting in a restricted parallax which may constrain the ants’ ability to match stored snapshots when approaching the nest from different directions. On the other hand, shorter learning walks due to spatial restrictions include fewer pirouettes possibly resulting in fewer stored snapshots. To build a stable long-term memory, repeated snapshots may be necessary, i.e., spatial restrictions during learning walks may result in an impaired memory about the landmark panorama.

When Australian jack jumper ants (*Myrmecia croslandi*) are displaced in their natural habitat within 10 m in various directions from the nest, they return to their nest directly (Narendra et al. 2013). The authors suggest that the ants have acquired sufficient information about the panorama close to the nest by taking snapshots, so that they can return from places to the nest where they may have never been before. However, since the experience of individual jack jumpers prior to testing was not known, the ants could have potentially visited the release sites before they had been experimentally transferred to them. In contrast, in the present study, individually marked ants had been recorded from their first foraging trip onwards. It, therefore, can be excluded that they have ever been at a release site before (see test paradigms FV I, FV 1st, and FV 10+, and Figs. 4, 5, 6). Depending on the space restrictions during the learning walks, in the PI versus LG competition experiments, the ants having initially followed their PI vector start either LG (under conditions of moat setup 2: Figs. 4b, 5b, e; moat setup 3: Figs. 4c, 5c, f) or perform systematic searches (under condition of moat setup 1: Figs. 4a, 5a, d). In conclusion, the spatial restrictions of the moat influenced the homing success of the ants significantly (Fig. 7). Under moat setup 1 conditions, learning walks are virtually prevented. This enables us to ask what landmark knowledge that the ants acquire with increasing number of foraging runs (feeder visits) alone. This is the kind of question to which we turn next.

### Gradual transition from path integration to landmark guidance

Learning the landmark panorama around the nest or any other goal is not a one-shot event. As shown in *C. fortis*, the ants must perform a suite of nest-centered learning walks, in which they move in loops of ever increasing size around the nest, until they are finally able to locate the goal accurately and precisely on the basis of landmark information alone (Müller 1984; Fleischmann et al. 2016). Hence, landmark memories—memories of panoramic views later used in returning to the nest—are acquired gradually. In contrast, PI works from the very start of an ant’s foraging career. As, in fully fledged foragers, LG can completely override the dictates of the path integrator (e.g., Andel and Wehner 2004), it is a likely hypothesis that early in an ant’s foraging life LG gradually gains in importance relative to PI. We have tested this hypothesis by exposing ants, which had just started their foraging lives, to cue-conflict situations, in which PI and LG information led the ant in opposite directions (different by 180°). As expected, all FV ants (trained in the free field or one of the experimental setups), which were displaced from the feeder to the release point, first followed the direction indicated by the PI home vector and then switched to LG or systematic search.

To test the influence of experience gained over time during training on the homing abilities during testing, ants were captured after different numbers of feeder visits. FV ants tested for the first time, i.e., displaced after they had arrived at the feeder on their first foraging trip in life, selected the PI direction, and reeled off their home vector to about 76–92% (FV I and FV 1st ants, Fig. 8) before switching to another navigational strategy. When being tested after at least ten feeder visits, ants already stopped following the PI vector after 52–66% of its length had been run off (FV VI and FV 10+ ants, Fig. 8). With an increasing number of feeder visits, ants stopped following their PI home vector even earlier as can be seen in the ants tested multiple times during their foraging career. Even though the homing behavior differed most obviously between ants being tested for the first and the second time (i.e., ants tested for the first time followed their PI home vectors almost completely, whereas ants tested repeatedly stopped earlier to follow their PI vectors), the distance between release point and turning point continued to decrease in subsequent tests (for individual examples: Fig. 4). Importantly, FV ants always followed their PI home vectors to some degree of their home vector length (as did the free-field experienced foragers, Fig. 2b). Therefore, landmark experience gained over time during multiple feeder visits gradually decreased the impact of PI guidance.

Homing success depends on the spatial experience gained during learning walks (as discussed above). There was an obvious behavioral difference between ants trained in the moat setup 1 and ants trained in the large moat setup 3. Ants that had previously been able to perform their learning walks in the large moat setup 3 setup, followed their home vector, turned around, exhibited some search behavior, and finally returned to the nest by LG. In contrast, ants that had not had space to perform learning walks prior to testing, also followed their home vectors, but then exhibited search behavior centered on the fictive position of the nest entrance and never returned to the actual nest area—not even after extensive training and several tests (Fig. 7). Therefore, extensive learning walks are necessary to enable ants to return to their nest by LG.

This conclusion is corroborated by ants that were tested as zero-vector (ZV) ants, which being devoid of PI information must rely exclusively on LG (see, e.g., Wehner et al. 1996; Kohler and Wehner 2005; Wystrach et al. 2012). Due to the experimental schedule applied in the present study, all ZV ants had been at the release point before as FV ants. Furthermore, when starting their searches, they were closer to the goal, i.e., the nest, than the FV ants. This is for the simple reason that the FV ants, while initially following their PI vector had moved away from the nest for about 7–10 m. Hence, it should have been easier for the ZV ants than for the FV ants to reach the nest, but this was not the case. In the moat setup 1 situation only 12 of 31 ZV ants returned to the nest [ZV (*n* = 10) and ZV10+ (*n* = 21)]. The success rate was high only in ants that have had enough space to perform learning walks (i.e., were trained in moat setup 2 and 3) before foraging and testing (Fig. 7). These ants did not return to the nest by systematic search behavior. Rather, they approached the nest directly (Fig. 4c ZV, Fig. 6c). Hence, the ZV tests confirm the results obtained in the FV tests that learning walks are necessary for successful homing.

Several studies performed in different desert ant species have investigated the ants’ navigational performance under PI-LG cue-conflict conditions (e.g., *Cataglyphis*: Wehner et al. 1996, see Fig. 11 therein; Wystrach et al. 2012, 2015; *Melophorus*:; Kohler and Wehner 2005; Legge et al. 2014; *Myrmecia*:; Freas et al. 2017; Narendra et al. 2013). However, only two recent studies focus particularly on the influence that increased experience has on the ants’ decisions (Freas and Cheng 2017; Schwarz et al. 2017). Both studies show that Australian red honey ants (*Melophorus bagoti*) rely more on LG as compared to PI, the more frequently they have travelled a familiar feeder-nest route, as shown in the present study for *C. noda*.

In the first study (Freas and Cheng 2017), ants were trained to forage within an arena (diameter: 2 m) around the nest entrance, with a feeder included in the arena. When naïve FV ants were displaced to a release point located in the opposite direction to the feeder at an 8 m distance outside of the arena, these ants did not orient in the true nest direction as indicated by terrestrial cues, but followed their PI vector. Experiencing the route from release point to the nest once during testing did not change this result when, thereafter, the ants were tested for the second time. However, after a training procedure of several feeder visits and transfers to the release point as ZV ants, FV ants oriented towards the true nest direction. Hence, information acquired during training caused overriding a conflicting PI vector. The second study (Schwarz et al. 2017) shows that repeated travels along a familiar route let landmark scenes distant from the route appear more unfamiliar than before route learning has started. As deduced from LG-PI 180° cue-conflict experiments, with increasing route training, a familiar scene becomes more readily distinguished from an unfamiliar scene. In particular, when naïve ants that were displaced to an unfamiliar distant test field after their first visit to a feeder (set up 8 m from the nest entrance), they followed their PI vector to about 80% before starting to search for the nest. In contrast, experienced ants that had visited the feeder for 2 days, ran off only about 40% of their PI vectors. These results are in principal accordance with the performance of the FV VI and FV 10+ ants in the present study.

In conclusion, differences in both spatial and temporal dimensions influence the navigational performance of *Cataglyphis* ants tested at different stages of experience. The ants need enough space to perform their learning walks around the nest entrance to later return to the nest reliably. More time for experiencing a foraging route reduces the impact of conflicting PI information. Therefore, both more space for performing learning walks and more time for repeatedly visiting a familiar site help ants to find back to the nest from places at which they have never been before. Comparison with similar results obtained in other desert ants indicates that using terrestrial cues for landmark guidance is a process that starts with the learning walks of novices and continues throughout the ants’ entire foraging lives.

## Abbreviations

FV: Full vector
FFFV: Free-field full-vector foragers
FFNO: Free-field novices
GCFV: Glass-channel full-vector ants
LG: Landmark guidance
PI: Path integration
ZV: Zero vector
